# Trivinylphosphine Oxide: Synthesis, Characterization, and Polymerization Reactivity Investigated Using Single-Crystal Analysis and Density Functional Theory

**DOI:** 10.3390/molecules28166097

**Published:** 2023-08-17

**Authors:** Khalifah A. Salmeia, Akef T. Afaneh, Reem R. Habash, Antonia Neels

**Affiliations:** 1 Department of Chemistry, Faculty of Science, Al-Balqa Applied University, Al-Salt 19117, Jordan; 51907039006@std.bau.edu.jo; 2 Laboratory for Advanced Fibers, Swiss Federal Laboratories for Materials Science and Technology (Empa), 9014 St. Gallen, Switzerland; 3 Center for X-ray Analytics, Swiss Federal Laboratories for Materials Science and Technology (Empa), 8600 Dübendorf, Switzerland

**Keywords:** organophosphorus, Michael-like addition, Grignard reagent, X-ray analysis, DFT calculations, reaction profile, kinetic and thermodynamic comparison

## Abstract

Organophosphorus chemicals are versatile and important in industry. Trivinylphosphine oxide (TVPO), for example, exhibited a promising precursor as a flame-retardant additive for industrial applications. Density functional theory (DFT) simulations were used to explore the kinetic and thermodynamic chemical processes underlying the nucleophilic addition reactions of TVPO in order to better understand their polymerization mechanisms. An experimental X-ray single-crystal study of TVPO supported this work’s theory based on its computed findings. TVPO was prepared using POCl_3_ and VMB in a temperature-dependent reaction. TVPO, the thermodynamically favourable product, is preferentially produced at low temperatures. The endothermic anionic addition polymerization reaction between TVPO and VMB begins when the reaction temperature rises. An implicit solvation model simulated TVPO and piperazine reactions in water, whereas a hybrid model modelled VMB interactions in tetrahydrofuran. The simulations showed a pseudo-Michael addition reaction mechanism with a four-membered ring transition state. The Michael addition reaction is analogous to this process.

## 1. Introduction

Organophosphorus compounds are one of the most important classes of organic chemistry due to their wide applications such as polymer additives in industry, acting as flame retardants [1,2] and antioxidants [3]. In general, the flame retardant efficacy of polymeric matrices can be enhanced using either reactive or non-reactive flame retardant additives [4,5]. Organophosphorus compounds can be classified according to their valency (λ) and coordination number (σ), affording for example: (i) P(V) pentavalent tetracoordinated compounds (λ^5^σ^4^), such as phosphine oxides, phosphoramidates, phosphonamidates, and phosphates; (ii) P(III) tervalent tricoordinated compounds (λ^3^σ^3^), such as phosphine, phosphites, and phosphorus trichloride, and (iii) P(III) pentavalent tetracoordinated compounds (λ^5^σ^4^), such as H-phosphonates and H-phosphinates [6]. Due to the importance of these compounds, different synthetic approaches have been explored using elemental phosphorus as a foundation for these approaches [7], and benign synthetic approaches have been also investigated [8,9]. The protection and deprotection of phosphine compounds using borane complexes are one of the numerous essential applications characterized by simple, effective, and environmentally friendly protocols for protecting the phosphorus atom with borane and then removing this protection from tertiary phosphines under benign conditions. Such reactions are analysed kinetically, and computational modelling facilitates the design of customized reaction conditions based on the substrate’s specific requirements. These advancements enhance the adaptability and utility of phosphine compounds for a wide range of applications [10].

**Figure 1:**
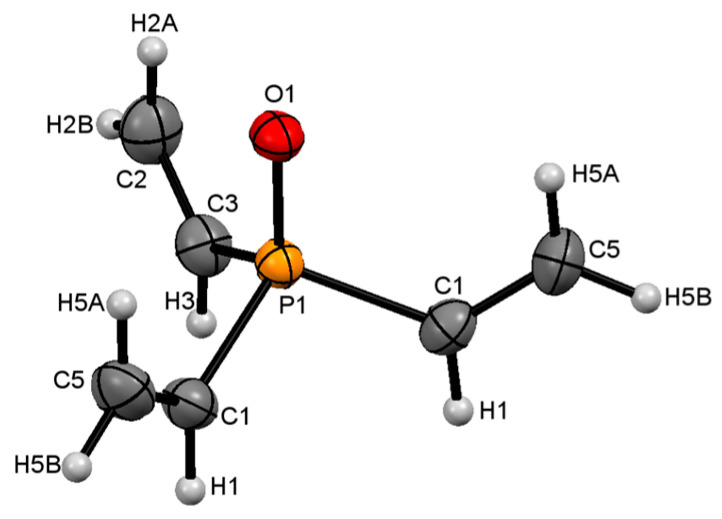
ORTEP crystal structure of TVPO showing atoms labelled at the 50% ellipsoidal probability level.

Among all of these synthetic approaches, the formation of P-C bonds to produce phosphine oxides has attracted special attention due to their thermal and chemical stability [11]. Amongst the phosphine oxide compounds, trivinylphosphine oxide (TVPO, Figure 1) has received interest due to its promising prospective potential applications. Thus, different synthetic approaches have been followed to synthesize TVPO [12,13,14,15,16]. The reactivity of the vinyl group towards radical polymerization and Michael-like addition reactions has been investigated using the reactive extrusion approach [17]. This methodology forges in situ preparation of polymeric material acting as immobilized flame-retardant additives. Such methodologies have been also applied for the preparation of durable flame retarded cellulose fabric materials using physical networks, with the polymerization of TVPO and piperazine at high-temperature [18]. Moreover, the Michael-addition reaction between TVPO and piperazine in water afforded pH-responsive nanostructured hydrogels with potential applications in drug release [19]. To the best of our knowledge, the nucleophilic addition reaction of α,β-unsaturated phosphine oxides using theoretical modelling has not been explored. The present study aims to investigate the synthesis of TVPO and examine its chemical reactivity with respect to Michael-like addition reactions and anionic addition polymerization reactions using a Grignard reagent. This is achieved with the application of thermodynamic and kinetic principles using theoretical modelling, including the corresponding molecular configurations. With the integration of experimental synthesis and single-crystal analysis with computational methodologies, significant insights into the reaction mechanism were obtained regarding the thermodynamic stability and kinetic behaviour exhibited by TVPO during its chemical reactions. The results of our study offer a thorough understanding of the reactivity exhibited by TVPO. This contributes to the development of knowledge in this particular area and creates opportunities for potential applications in the fields of materials science and polymer chemistry.

## 2. Results and Discussion

### 2.1. Synthesis and Structural Analysis

In this study, TVPO was synthesized with the reaction of vinylmagnesium bromide (VMB) and phosphoryl trichloride in a 3 to 1 molar ratio at −78 °C. The reaction product is temperature-dependent, and an increase in the reaction temperature results in the formation of a bulk precipitate that is attributed to the in situ polymerization of TVPO [16]. Hence, we investigated the kinetic and thermodynamic product selectivity of the reaction between POCl_3_ and VMB. Theoretical calculations indicate that the reaction between POCl_3_ and VMB is both thermodynamically favourable and exhibits rapid kinetics. The reaction proceeds through a nucleophilic substitution mechanism, in which the vinyl anion derived from VMB acts as the nucleophile and attacks the phosphorus atom of POCl_3_. The first step in the process involves attacking the phosphorus atom with a vinyl anion, which causes the displacement of a chloride ion. This step is fast, due to the high reactivity of the vinyl anion as a nucleophile and is accompanied by an exothermic process that results in an energy release of 61 kcal/mol. Afterwards, a vinyl anion from VMB reacts at a fast rate with the intermediate species’ phosphorus atom (vinylphosphonic dichloride), causing another chloride ion to be displaced, thereby releasing 53 kcal/mol of energy. The reaction progresses through a series of swift nucleophilic substitution steps, ultimately resulting in the formation of TVPO that releases 160 kcal/mol of energy in total. The rate of a reaction can be affected by various factors, including temperature, the concentration of reactants, and the properties of the solvent. Overall, the reaction between POCl_3_ and VMB is characterized by rapid kinetics and thermodynamic favourability. This is attributed to the high reactivity of the vinyl anion and the favourable conditions for nucleophilic substitution. The analysis conducted indicates that the vinyl anion derived from VMB displays significant reactivity, which facilitates the reaction with TVPO. This reaction leads to the formation of polymers, as explained in the section covering the anionic addition reaction using a Grignard reagent. The chemical structure of TVPO was confirmed using NMR spectroscopy and single-crystal analysis, and it was found to be identical to published reports. Combining the single-crystal data from our analysis and a previous report [15] allowed us to validate the level of theory used in this study based on bond lengths and the nature of inter- and intra-molecular interactions. A summary of the crystal data and structure refinement are summarized in Table 1 and the Supplementary Materials (Tables S1–S5).

From the crystal structure, the bond length of P−O is found to be 1.486 (2) Å. This bond length is shorter than the sum of the single-bond radii of phosphorus (1.07 Å) and oxygen (0.66 Å) atoms. Thus, the P−O bond is shorter than the single bond, which indicates a double bond nature. However, the short bond length of P−O can also be attributed to the attraction nature of the P^+^O^–^ bond [20,21]. The validation of our modelling approach used herein was performed by matching the experimental bond lengths of TVPO (Figure 1), as revealed using single-crystal X-ray analysis (of two different crystals), with the bond lengths, which were calculated using our level of theory summarized in Table 2.

**Figure 2:**
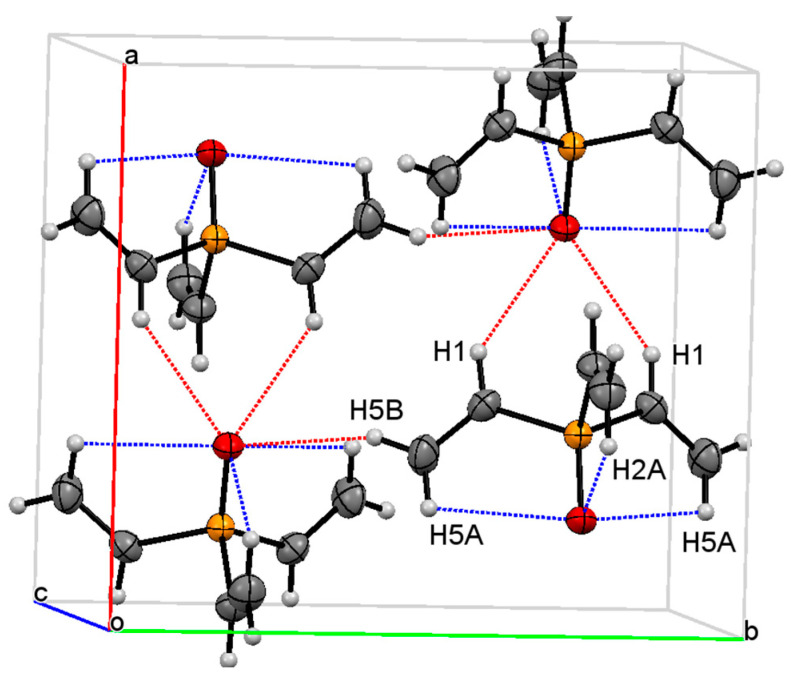
Packing diagram showing the ORTEP structure for TVPO per unit cell with a 50% ellipsoidal probability level. P: yellow; O: red; C: grey; H: white. Blue dotted lines represent intramolecular O···H bonds, while red dotted lines represent intermolecular O···H bonds.

The bond lengths for both crystal structures, as listed in Table 2, are consistent with the characteristics of the chemical bonds and agree with the results of the single-crystal study. The existence of four TVPO molecules per unit cell was discovered during an investigation of a single crystal (Figure 2).

The order of the atom orientation in the structure packing reveals the nature of the inter- and intra-molecular interactions among the atoms and shows the probability of electron density within the molecule. Within each molecule (Figure 2), the oxygen atom has intramolecular contacts with H5A and H2A hydrogen atoms of =CH_2_ groups (blue dotted lines; bond lengths are summarized in Table 2). In addition, other hydrogen atoms are involved in intermolecular contact with oxygen atoms of neighbouring molecules (red dotted lines) Therefore, the electron density is more likely concentrated on the oxygen atoms of TVPO.

### 2.2. DFT Calculations

The initial stage of our discourse entails meticulous scrutiny of the TVPO configuration derived from our computations conducted within the M062X/6-311++G(d,p) theoretical framework. The aim of this study is to evaluate the reliability and accuracy of the selected theoretical framework using a comparative analysis of the computational outcomes and the X-ray data obtained from experiments. The present comparative study aims to offer significant insights into the accuracy and validity of our theoretical approach to elucidating the intricate molecular architecture of TVPO. The outcomes of this assessment will establish a basis for making a knowledgeable decision regarding the appropriateness of using the M062X/6-311++G(d,p) level of theory to investigate the reaction profiles of TVPO in two separate classifications: nucleophilic and Grignard addition reactions.

Within the realm of our current knowledge, two rigorous X-ray studies were conducted to unravel the intricate structure of TVPO, as outlined in Table 2. These investigations yielded compelling evidence, suggesting the presence of a pseudo C_3v_ symmetry encircling the principal rotational axis. Notably, the measured P–O bond length obtained from these studies exhibits a range spanning from 1.489 to 1.500 Å. In the pursuit of deeper comprehension, our research endeavours extended to predictive analysis. Using meticulous calculations, we determined a predicted bond length of 1.503 Å (Table 2). While this value modestly exceeds the experimental range, it remains within close proximity, highlighting the significant alignment between theoretical expectations and empirical observations. Furthermore, Table 2 presents additional noteworthy findings regarding the P–C bond length in TVPO. Specifically, the X-ray measurements (Table 2) demonstrate a range of 1.787–1.796 Å, while our present study yields a value of approximately 1.790 Å. The remarkable agreement between our results and experimental data serves as a strong validation of our research methodology and underscores the robustness of our findings. Expanding our analysis to encompass C–C bond lengths and the intramolecular O···H distances, the M062X/6-311++G(d,p) optimization approach was used. As indicated in Table 2, the predicted C–C bond lengths range from 1.314 to 1.320 Å, while the O···H distances span from 2.731 to 2.743 Å. Remarkably, these calculated ranges align remarkably well with the corresponding experimental values of 1.310–1.321 Å and 2.706–2.780 Å, respectively. This convergence serves to bolster our confidence in the predictive capability of the used level of theory, accentuating its profound utility in elucidating the intricate complexities within the realms of two distinct reaction profile categories.

Nucleophilic addition reactions are a crucial category of conversions in organic synthesis. The exploration of the reactivity of TVPO at the critical β-C position (of the vinyl group) provides opportunities for innovative synthetic methodologies. The present investigation is centred on the examination of the nucleophilic addition reactions of TVPO with piperazine (i.e., pseudo-Michael addition) [22,23] and VMB (i.e., anionic addition reaction using a Grignard reagent) reactions. Our objective is to elucidate the complex thermodynamic and kinetic aspects of every reaction through the determination of the free energy and barrier heights for both the forward and reverse directions.

#### 2.2.1. Nucleophilic Addition Reaction (Pseudo-Michael Addition) Using Piperazine

**Scheme 1:**
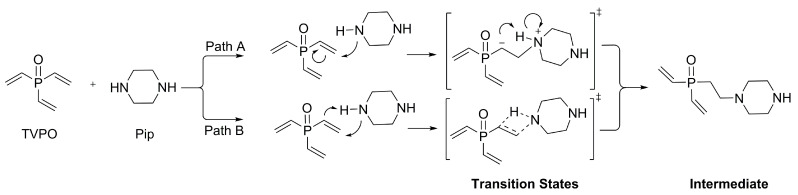
Proposed reaction mechanism between TVPO and piperazine, illustrating two plausible reaction pathways (Path A and Path B).

The chemical reaction between TVPO and piperazine has garnered considerable interest owing to their multifaceted utility [17,18,19]. A plausible reaction mechanism between TVPO and piperazine (Pip) was proposed in this work to identify the possible reaction pathway (Scheme 1).

**Figure 3:**
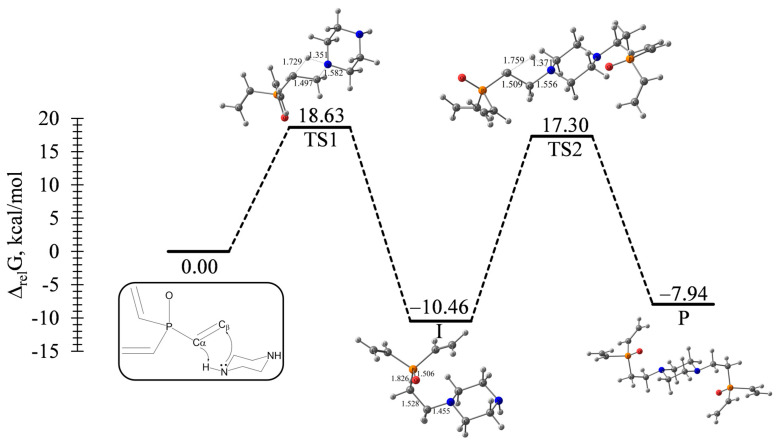
The potential energy diagram for the reaction between TVPO and piperazine. The energies were calculated at the M062x/6-311++G(d,p) level of theory.

The present investigation aims to examine the two-step reaction mechanism that culminates in the creation of the adduct. Our study places particular emphasis on the intermediate (2-(piperazin-1-yl)ethyl)divinylphosphine oxide monoadduct (Scheme 1, Intermediate). Based on a comprehensive examination, it is suggested that the monoadduct plays a fundamental role as an intermediary between two transitional states, thereby providing insight into the fundamental reaction mechanism. The chemical process occurs via a biphasic mechanism, commencing with the generation of a preliminary pre-reactive amalgamation, designated the monoadduct intermediate (I), via the transition state TS1. Within TS1 (Figure 3), a hydrogen (H) atom is situated in a poised position between the terminal nitrogen atom of piperazine and the carbon, thereby effectively creating a bridge as visually depicted in Figure 3 and Scheme 1 (Path B).

The extension of the bond length may arise from the establishment of a bridging interaction linking the nucleophilic nitrogen atom and the β-carbon (Cβ). The process underlying the bridging interaction entails the exchange of electron density and the adjustment of bond lengths to facilitate the formation of a novel bonding configuration. It is noteworthy that the initial product resulting from the reaction between TVPO and piperazine in a 1:1 mole ratio is the (2-(piperazin-1-yl)ethyl)divinylphosphine oxide monoadduct (I). The results of this study indicate that the reaction proceeds in a consistent manner, culminating in the creation of the intermediate, even when the ratio is 2:1. Following this, the (2-(piperazin-1-yl)ethyl)divinylphosphine oxide monoadduct (I) undergoes a transformation towards the intended (piperazine-1,4-diylbis(ethane-2,1-diyl))bis(divinylphosphine oxide) adduct (P) via transition state TS2, which exhibits a comparable H-bridge arrangement. Figure 3 and Scheme 1 (Path B) illustrate the prevalence of a four-membered ring in both TS1 and TS2.

In the transition state TS1, a distinct geometric transformation becomes evident. Notably, the N-Cβ bond exhibits a discernible elongation of approximately 0.127 Å compared with the corresponding bond length in the monoadduct. Simultaneously, the Cα-Cβ bond experiences a contraction by approximately 0.031 Å in the transition state relative to the monoadduct. Furthermore, the N and Cβ atoms come closer to each other, resulting in a distance of 1.582 Å between them. It is noteworthy that this bond formation occurs at a length of 1.455 Å in the (2-(piperazin-1-yl)ethyl)divinylphosphine oxide monoadduct intermediate (I), thus indicating a progression towards the transition state. In the transition state TS2, as depicted in Figure 3, notable changes in bond lengths are observed. The Cα-Cβ bond demonstrates a minor elongation of 0.012 Å in comparison with the corresponding bond length observed in TS1. In contrast, the Cβ-N bond experiences a contraction of 0.026 Å, suggesting a more condensed configuration. Furthermore, it is noted that the distances separating the H-bridge, the Cα, and N atoms exhibit greater lengths in TS2 in comparison to TS1. This observation implies that the configuration of TS2 exhibits a greater level of relaxation, potentially suggesting a reduced energy barrier in the reaction pathway in comparison with TS1.

The thermodynamic and kinetic favourability of a chemical reaction are essential considerations that significantly contribute to comprehending its feasibility and rate of occurrence. The present study aimed to examine the reaction depicted in Figure 3 and assess its thermodynamic and kinetic properties. The activation energies (*Ea*) for the first and second steps were found to be 18.63 kcal/mol and 17.3 kcal/mol, respectively. The *Ea* observed in the second step implies a higher degree of kinetic favourability, thereby indicating a comparatively accelerated reaction rate in comparison to the first step. The computed values for the free energy changes indicate that the reaction exhibits exothermic properties, consistent with the findings of our investigation. The free energy alterations associated with the creation of the monoadduct, adduct, and bis-adduct products are 10.46 kcal/mol, 7.94 kcal/mol, and 8.40 kcal/mol, correspondingly. Exothermic reactions generally demonstrate thermodynamic favourability as they release energy and progress towards a more stable state in relation to Gibb’s free energy (∆G). Based on the provided activation energies and the exothermic nature of the reaction, it can be deduced that the reaction is thermodynamically and kinetically favourable. This statement implies that the reaction exhibits a high degree of reactivity and is exothermic in nature. Furthermore, a comprehensive examination of the entropy (∆S) values of the products was carried out. The entropy values of three different compounds were measured. The first compound, (2-(piperazin-1-yl)ethyl)divinylphosphine oxide monoadduct, has an entropy value of 125.733 cal/mol·K. The second compound, (piperazine-1,4-diylbis(ethane-2,1-diyl))bis(divinylphosphine oxide) adduct, has an entropy value of 173.843 cal/mol·K. The third compound, TVPO-PIP-TVPO-PIP bisadduct, has an entropy value of 199.377 cal/mol·K. The values of ∆S mentioned here offer further evidence for the thermodynamic favourability of the reaction. This is because an increase in entropy is usually linked to reactions that are more favourable.

Determining the rate constants for the two reaction steps is of great significance, considering their magnitudes. In this study, we used the Eyring–Polanyi equation, a comprehensive framework that accounts for both temperature and energy barrier (*Ea*) effects. The Eyring equation, given by k = (k_b_T/h)exp(−ΔG^‡^/(RT)), characterizes the rate constant (k) of the reaction [10,24]. Here, k_b_ represents the Boltzmann constant (1.380649 × 10^−23^ J/K), T denotes the absolute temperature in Kelvin, h signifies the Planck constant (6.62607015 × 10^−34^ J·s), ΔG^‡^ corresponds to the ∆G of activation, and R stands for the gas constant (8.314 J/(mol·K)). Our comprehensive computational analysis discovered an extraordinary ratio rate constant of 9.5 for the sequential addition of TVPO to (2-(piperazin-1-yl)ethyl)divinylphosphine oxide monoadduct, culminating in the synthesis of (piperazine-1,4-diylbis(ethane-2,1-diyl))bis(divinylphosphine oxide) adduct. The determination of *Ea* values, specifically, 17.3 kcal/mol for the second addition and 18.63 kcal/mol for the first addition, yields insights into the dynamics of reaction kinetics and the underlying mechanisms, as depicted in Figure 3 and Scheme 1 (Path B). This pivotal research serves as a cornerstone for future explorations aimed at unravelling the intricacies of selectivity and reactivity within complex multi-step reactions.

#### 2.2.2. Anionic Addition Reaction Using a Grignard Reagent

**Scheme 2:**
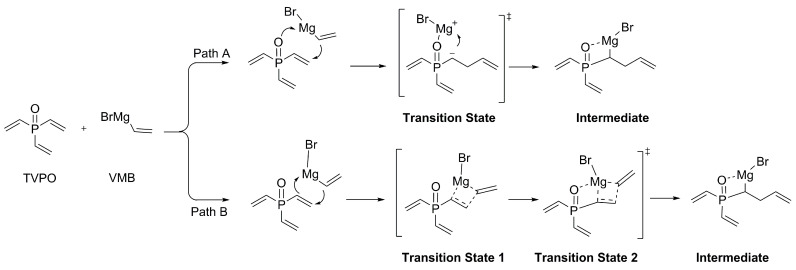
Proposed reaction mechanism between TVPO and VMB, illustrating two plausible reaction pathways (Path A and Path B). Coordinated solvents were omitted for clarity.

TVPO has the potential to undergo an anionic addition mechanism upon its reaction with the Grignard reagent vinylmagnesium bromide (VMB). The reaction process involves the application of VMB as a nucleophile, which triggers an attack on the electrophilic carbon (Cβ) of TVPO. Two plausible reaction mechanisms between TVPO and VMB have been proposed (Scheme 2) to identify the possible reaction pathway using the mononuclear Grignard reagent species [25].

The reported crystal structures of the Grignard reagents exhibited their existence in monomeric, dimeric, and tetrameric structures in the solid state. However, their solid-state structures may not be representative of their active species in solution. The monomeric structure of Grignard reagents in solution was reported to exist in a thermodynamic equilibrium with their corresponding complex species, and hence, VMB was used in its simple solvated molecular structure. To ensure accurate modelling of VMB, we decided to use its simple solvated molecular structure, taking into account the equilibrium involved [26,27,28]. The process involves the creation of an intermediate chemical species, which then undergoes protonation or additional reaction pathways, affording the final product.

**Figure 4:**
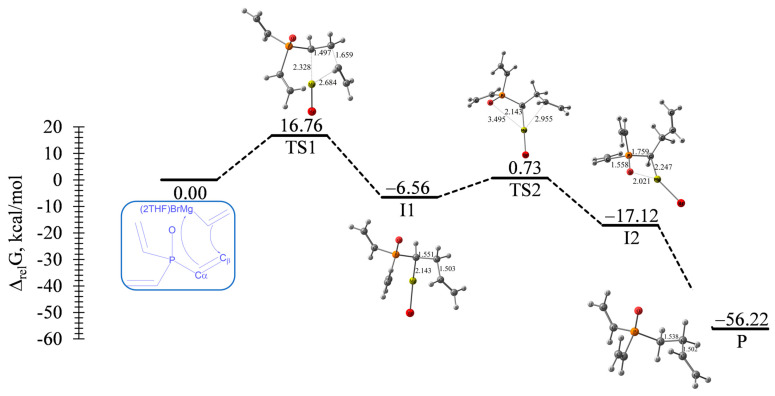
The potential energy diagram for the reaction between TVPO and VMB.2THF. The energies were calculated at the M062x/6-311++G(d,p) level of theory.

Clarifying our original investigation is crucial for gaining a better understanding of the mechanisms underlying the TVPO and VMB reactions (Figure 4). The primary goal of this study is to determine where MgBr and TVPO interact most commonly when the CH_2_CH^−^ nucleophile attacks TVPO’s Cβ and to investigate if the interaction takes place mostly with the Cα or the oxygen atom (Scheme 2). To undertake a complete analysis, we focused on three key elements: the distribution of natural bond orbital (NBO) charges during carbanion formation, geometrical analysis, and the resulting stabilization energy (E2) that arises from orbital interactions between species. According to our findings, the NBO charge of the Cα in TVPO is −0.575, while the charge of the 1-(divinylphosphoryl)but-3-en-1-ide anion (V-TVPO) is around −1.055. The oxygen atom in TVPO, on the other hand, has an NBO charge of −1.135. However, in the V-TVPO anion, it rises insignificantly to −1.210. Based on these data, the charge density is most likely carried by the Cα atom, indicating its nucleophilic affinity more than the oxygen atom. The HOMO molecular orbitals of both TVPO and the V-TVPO anion are designated as the lone pair of oxygen atoms (Lp_O_) and the lone pair of carbon atoms (Lp_Cα_). The geometrical analysis of TVPO and the V-TVPO anion reveals that the Cα-P bond in the V-TVPO anion is greatly shortened by about 0.08 Å, while the P-O bond is somewhat lengthened by approximately 0.02 Å. This shortening is caused by the absence of the π_P-Cα_ bond in TVPO, which is, on the other hand, formed in V-TVPO due to the 9% contribution of the phosphorus d-orbital. Finally, the Fock matrix analysis using second-order perturbation theory in the NBO basis shows that the stabilization energies (E2) for C···Mg (Scheme 2, Path B) and O···Mg (Scheme 2, Path A) are 42 kcal/mol and 16 kcal/mol, respectively. The greater possibility of bonding between MgBr and the Cα atom, due to its higher nucleophilicity, has a significant impact on the system’s reactivity. As a result, the Cα atom plays an important role as a strong nucleophile, exceeding the oxygen atom in its ability to form a coordination bond with MgBr.

For all species represented in Figure 4, the hybrid solvation technique, which combines implicit and explicit solvation models, was used. We obtained significant results in our investigation into the role of tetrahydrofuran (THF) as a solvent in connection to the Grignard reagent (Supplementary Materials, Figure S2). THF, for example, was shown to stabilize the Grignard reagent by ca. 11.8 kcal/mol. A detailed NBO analysis also revealed that electrostatic forces are the primary mode of interaction between THF and the magnesium (Mg) metal ion. It is important to note that this interaction has neither covalent nor coordination bonds; therefore, no matching frequency was observed. Furthermore, the closest separation distance between the two THF molecules and the matching species, according to the proximity analysis, as depicted for the intermediate I1 is around 4.6 Å (Supplementary Materials, Figure S3). As a result, the presence of the solvent has no influence on these species’ energy. Therefore, we determined the reaction profile of TS1, TS2, I1, and I2 species (Figure 4) in the absence of the THF solvent molecules (excluding the Grignard reagent). Based on these observations, the overall pathway of the reaction profile did not change in comparison to the implicit solvation model (Supplementary Materials, Figure S4). It is worth noting that the energy of all the species included changed by 11.8 kcal/mol, together with TS1, TS2, I1, I2, and P (Figure 4, and Supplementary Materials, Figure S4). We also investigated the coordination of magnesium with the oxygen atom. Our findings show that the resulting model molecule (Supplementary Materials, Figure S5) is approximately 6.5 kcal/mol less stable than intermediate I1 (Figure 4). Accordingly, the chemical properties of TVPO and α,β-unsaturated ketones must be compared: the P-atom hybridization of TVPO is sp^3^d, whereas the carbonyl carbon in α,β-unsaturated ketones is sp^2^. Variations in hybridization may affect the resonance of conjugated double bonds due to the spatial configuration of valence molecular orbitals. Furthermore, the chemical reaction between Grignard reagents and α,β-unsaturated ketones results in the production of alcohols via the 1,2-addition process [29]. In contrast, Grignard reagents undergo a Michael-like addition reaction with TVPO, which is similar to the 1,4-addition reaction between Gilman reagents and α,β-unsaturated ketones, which results in the formation of a Michael-addition product. However, depending on the specific characteristics of the Gilman reagents and the stereochemistry of the enones under consideration, multiple reaction routes have been found [30,31]. After the detailed explanation provided above, we can now proceed to outline the mechanism underlying the TVPO and VMB reaction based on these findings.

The initiation of the chemical interaction between TVPO and VMB occurs through a transitional state, which involves the formation of a four-membered ring that includes Mg and the TVPO molecule (Figure 4 and Scheme 2, Path B). The transition state under consideration displays an *Ea* of 16.76 kcal/mol and leads to the formation of an intermediate chemical species. In the transition state, a chemical bond is formed between the magnesium atom and the Cα, with a bond length of 2.328 Å. Additionally, the vinyl group is bonded to the Cβ of the TVPO molecule, with a bond length of 2.684 Å, as depicted in Figure 4. The configuration mentioned above results in the formation of an intermediate with considerable stability, as evidenced by a stabilization energy of −6.56 kcal/mol. The bond lengths of Mg–Cα, Cβ–C=C and Cα-Cβ were observed to relax by 2.143 Å, 1.503 Å, and 1.551 Å, respectively, as shown in Figure 4. Subsequent reactions take place after the intermediate stage, ultimately leading to the formation of the desired final product known as but-3-en-1-yldivinylphosphine oxide (P). The synthesis of this specific chemical species is accompanied by the release of 56.22 kcal/mol of energy through an exothermic reaction. The conformational arrangement of but-3-en-1-yldivinylphosphine oxide depends on the specific disposition of its constituent atoms and the stereochemical parameters that regulate its chemical reactivity. Acknowledging this aspect holds significant importance. The outcome is likely to be impacted by the interrelated nature of these variables.

In this work, the computational analysis of the rate constant associated with the reaction between TVPO and VMB reveals a rate constant of 78 M^−1^s^−1^, shedding light on the kinetics of this chemical process and providing valuable insights. One crucial aspect of our investigation revolves around the determination of *Ea* values. We calculated an *Ea* of 16.76 kcal/mol, which highlights the intricate dynamics governing the reaction kinetics and offers a deeper understanding of the underlying mechanisms involved. Figure 4 illustrates detailed representations of these mechanisms, thereby enhancing our comprehension of the system. Significantly, the formation of the intermediate occurs through an exothermic reaction, liberating 6.56 kcal/mol of energy. The intermediate mentioned is important in the reaction pathway because it converts into intermediate I2 through TS2. The activation energy (*Ea*) for this conversion is 0.73 kcal/mol. TS2 is facilitated by the rotation around the P-Cα bond, which aligns the oxygen atom in a position corresponding to that of magnesium. As a result, a magnesium bridge is formed between the Cα and oxygen atoms, leading to the creation of I2. This new compound is more stable than I1 by 10.6 kcal/mol. The stable intermediate is crucial in the reaction pathway because it undergoes a direct transformation into the desired product but-3-en-1-yldivinylphosphine oxide (P). The process of conversion involves a loss in energy of 39 kcal/mol, indicating that the overall transformation is exothermic in nature.

By elucidating the rate constant, *Ea* values, and energy changes throughout the reaction, our study offers substantial insights into the details of this chemical process. These findings not only deepen our understanding of reaction kinetics but also bear significant implications for the development and optimization of synthetic methodologies involving the reaction between TVPO and VMB. The combination of a high rate constant and relatively low activation energy suggests that the reaction between TVPO and VMB is kinetically favourable. Additionally, the exothermic nature of the overall transformation indicates that the formation of the final product is thermodynamically favoured, with a substantial energy release. These factors further emphasize the feasibility and potential utility of this reaction in synthetic chemistry.

## 3. Materials and Methods

### 3.1. Materials

Vinylmagnesium bromide (1M in THF) and phosphoryl trichloride (POCl_3_, Reagent Plus, purity = 99%), ammonium chloride (NH_4_Cl, ACS reagent, purity ≥ 99.5%) and dry THF were purchased from Merck (Buchs, Switzerland). All Chemicals were used as received without further purification. TVPO was synthesized as previously reported with slight modifications at the large scale [16,19].

### 3.2. NMR Spectroscopy

The ^1^H, ^13^C, and ^31^P NMR data were recorded using a Bruker (Billerica, MA, USA) 400 MHz NMR spectrometer (Supplementary Materials, Figure S1). Chemical shifts for ^1^H NMR and ^13^C NMR chemical shifts (δ) in ppm were calibrated to the residual solvent peak (CDCl_3_: δ = 7.26 and 77.16 ppm, respectively). The chemical shift for ^31^P NMR was referenced to an external sample with neat H_3_PO_4_ (δ = 0.0 ppm).

### 3.3. Single-Crystal X-ray Analysis

Single crystals of C_6_H_9_OP (TVPO) were obtained from its solution in ethanol using slow solvent evaporation. A suitable crystal was selected and mounted on an ‘STOE IPDS-II’ diffractometer (Darmstadt, Germany). The crystal was kept at 173 K during data collection. Using Olex2 [32], the structure was solved with the olex2.solve [33] structure solution program using charge flipping and refined with the ShelXL [34] refinement package using least squares minimization. The crystal structure was deposited to CCDC under the number 2260460. The same crystal structure was deposited previously (deposition number 1277428) and used for comparison [15].

### 3.4. Computational Methods

The current investigation involved the optimization of structures using the density functional theory (DFT) methodology with the M062X functional, which was implemented using Gaussian 09 software [35]. The computations were carried out using the 6-311++G(d,p) basis set. Several strong considerations support the choice of the M062X/6-311++G(d,p) level of theory. The M062X functional has been shown to possess the capacity to produce accurate results for a wide variety of molecular properties, including geometries, energies, and reaction barriers. This functional has been thoroughly investigated and found to be effective in analysing reactions and complex systems. Furthermore, the use of the 6-311++G(d,p) basis set provides a significant and adaptable option, encompassing both polarization and diffuse functions. These qualities allow it to effectively account for electron correlation and adequately address the effects of electron density inside the system under examination. Therefore, it can be argued that the M062X/6-311++G(d,p) level of theory achieves an ideal equilibrium between precision and computing expenditure. While more advanced techniques may provide higher levels of computational cost, their processing requirements make them less feasible for larger systems. Given these circumstances, the selected theoretical level demonstrates a prudent balance, effectively meeting the research criteria without imposing an undue computational load [36,37]. The use of the conductor-like polarizable continuum model (CPCM) [38,39,40] was implemented at identical theoretical levels to accommodate for the impact of solvent effects. The simulations explored two alternative solvation models: an implicit solvation model using water solvent parameters for piperazine and a hybrid solvation model using tetrahydrofuran (THF) both explicitly and implicitly for BrMgC_2_H_3_ (VMB). The solvents in this study (water for the reaction between TVPO and piperazine and THF for the reaction between TVPO and VMB) were chosen based on their experimental use for each corresponding reaction [19]. Radii collected from the universal force field (UFF) were used to build the solute cavity. Frequency calculations were performed in order to verify the correct number of imaginary frequencies and ensure the precision and dependability of the crucial stationary points. Moreover, the IRC [41] methodology based on intrinsic coordinates was used to validate the energy profiles that connect the notable transition structures to their corresponding local minima. The optimized structures were subsequently used for single-point calculations using the M062X/6-311++G(d,p) level of theory. Gibb’s free energies (∆G) corresponding to the aforementioned M062X/6-311++G(d,p) calculations were determined based on the obtained single-point energies. Using a rigorous computational methodology that incorporated dependable theoretical frameworks and solvation phenomena, the precision and coherence of our computations were guaranteed, thereby furnishing significant insights into the thermodynamic characteristics and reaction mechanisms being scrutinized.

### 3.5. Synthesis of Trivinylphosphine Oxide (TVPO)

In a 3 L double-jacket reactor fitted with a mechanical stirrer, addition funnel, and nitrogen inlet, POCl_3_ (51.1 g, 0.33 mol) was charged under N_2_ flow. Dry THF (1000 mL) was then added under N_2_, and the reaction was cooled to −78 °C. A vinylmagnesium bromide solution in THF (1 M, 1 L, 1 mol) was then added dropwise. After complete addition, the reaction was stirred at −78 °C for 3 h. Cold (4 °C) ammonium chloride solution (100 mL, [NH_4_Cl] = 4 M) was then added, and the mixture was stirred for 5 min. While still cold, the precipitate was separated using filtration, affording a solution of two layers. The THF layer (upper layer) was separated, and the volatiles were completely removed in vacuum. The aqueous layer was then extracted with chloroform (2 × 500 mL and then 2 × 250 mL). The chloroform phase and the residue from THF layer were mixed and dried over anhydrous Na_2_SO_4_. Volatiles were removed in vacuum, affording a pale-yellow solid that was further dried in vacuum oven over night at 50 °C. After drying, the solid became white. The purity of TVPO was investigated using NMR spectroscopy, which showed a satisfying purity. Hence, the product could be used without any further purification process. Yield (32.0 g, 75%); m.p: 98–100 °C; ^1^H NMR (400 MHz, CDCl_3_) δ (ppm): 6.07–6.31 (m, 9H); ^13^C NMR (100 MHz, CDCl_3_) δ (ppm): 134.03 (s, =***C***H_2_), 130.46 (d, ^1^*J*_CP_ = 100 Hz, =***C***H); ^31^P{^1^H} NMR (162 MHz, CDCl_3_) δ (ppm): 17.45.

## 4. Conclusions

In conclusion, during the synthesis and derivatization of TVPO, it is crucial to control the purity of the polymerization processes when seeking industrial applications. This comprehensive computational analysis offers valuable insights into the intricate molecular architecture of TVPO and the reaction profiles of TVPO in Michael-like (pseudo-Michael) addition reactions and anionic addition polymerization reactions. This study showcases the dependability and precision of the M062X/6-311++G(d,p) theoretical model using a comparative evaluation with empirical X-ray information. The findings indicate a noteworthy correspondence between the theoretical predictions and empirical observations with regard to bond lengths, thereby confirming the methodology and outcomes.

The investigation of nucleophilic addition reactions with piperazine elucidated a mechanism consisting of two distinct steps, which entail the creation of an intermediate and the transition between states. The lower *Ea* values seen for the second step as well as the exothermic nature of the reaction are proof of its thermodynamic and kinetic favourability. The entropy values that were computed also suggest favourable circumstances. This study yields a more profound understanding of the reaction kinetics and the function of transitional states, thereby augmenting forthcoming research on specificity and responsiveness in multi-phase reactions.

This investigation pertains to the anionic addition polymerization reaction using the Grignard reagent VMB. This study reveals the emergence of a four-membered ring intermediate and consequent reactions that culminate in the production of the intended outcome. The exothermic reaction displays a high rate constant and comparatively low *Ea*, implying its kinetic favourability. The results of this study provide insight into the kinetics and mechanisms underlying the reaction, highlighting its viability and potential usefulness.

In order to have a comprehensive grasp of the sequential processes involved in a mechanism, it is important to conduct a meticulous reassessment of the essential elements relating to the species involved in the reaction. These critical elements include a comparison of geometries, electron density distributions, and stability energies associated with orbital interactions between different species. This thorough method is highly recommended as a general practice for analysing the mechanism underlying any response, both before and after its occurrence, at each individual stage within the mechanism. This systematic examination promotes a greater understanding of the underlying processes and gives dynamic insights into the complexities of chemical reactions by examining all possible interactions.

In general, this study enhances our understanding of the reactivity of TVPO, elucidates the underlying reaction mechanisms, and identifies prospective synthetic utilities. The integration of computational analysis, theoretical framework, and experimental validation augments our understanding of reaction profiles and establishes a foundation for forthcoming advancements and refinements in the realm of synthetic chemistry.

## Figures and Tables

**Table 1:** Crystal data and structure refinement of TVPO.

Identification Code	TVPO (CCDC 2260460)	TVPO (CCDC 1277428) [15]
Empirical formula	C_6_H_9_OP	C_6_H_9_OP
Formula weight	128.10	128.10
Temperature (K)	173.15	143
Crystal system	Orthorhombic	Orthorhombic
Space group	Pnma	Pnma
*a* (Å)	9.4940 (7)	9.5130 (2)
*b* (Å)	10.7754 (12)	10.7830 (3)
*c* (Å)	7.0590 (6)	7.0590 (3)
α (°)	90	90
β (°)	90	90
γ (°)	90	90
Volume (Å^3^)	722.15 (11)	724.10 (4)
Z	4	4
ρ_calc_ (g/cm^3^)	1.178	1.175
μ (mm^−1^)	0.286	0.286
F(000)	272.0	272.0
Crystal size (mm^3^)	0.5 × 0.3 × 0.2	0.5 × 0.20 × 0.15
Radiation	MoKα (λ = 0.71073)	MoKα (λ = 0.71073)
2Θ Range for data collection (°)	6.9 to 51.168	3.45 to 27.60
Index ranges	−10 ≤ h ≤ 11 −12 ≤ k ≤ 13 −8 ≤ l ≤ 8	0 ≤ h ≤ 12 0 ≤ k ≤ 14 0 ≤ l ≤ 8
Reflections collected	2929	25094
Independent reflections	723 [R_int_ = 0.0424, R_sigma_ = 0.0240]	839 [R_int_ = 0.036]
Data/restraints/parameters	723/0/437	839/0/65
Goodness-of-fit on F^2^	1.213	1.092
Final R indexes [I ≥ 2σ (I)]	R_1_ = 0.0275, wR_2_ = 0.0673	R_1_ = 0.0353, wR_2_ = 0.1030
Final R indexes [all data]	R_1_ = 0.0327, wR_2_ = 0.0855	R_1_ = 0.0355, wR_2_ = 0.1032
Largest diff. peak/hole (e Å^−3^)	0.22/−0.44	0.258/−0.305

**Table 2:** Exploring selected TVPO bond lengths. The calculated (aqueous phase) values with experimental measurements from this work ^a^ and other studies ^b^.

	CCDC 2260460 ^a^	CCDC 1277428 ^b^	Calculated, This Study ^c^
P−O	1.486 (2)	1.5004 (13)	1.503
P−C1	1.7886 (18)	1.796 (2)	1.792
P−C3	1.787 (3)	1.7929 (14)	1.791
C1−C5	1.310 (3)	1.316 (3)	1.314
C2−C3	1.313 (4)	1.321 (2)	1.320
O···H2A	2.7165 (15)	2.78 (4)	2.743
O···H5A	2.7065 (8)	2.75 (3)	2.731

^a^ This work. ^b^ Ref. [15]. ^c^ Calculated values using the M062X/6-311++G(d,p) level of theory.

## Data Availability

All data are available in this article.

## Supplementary Materials

The following supporting information can be downloaded at: https://www.mdpi.com/article/10.3390/molecules28166097/s1, Figure S1: NMR spectra of trivinylphosphine oxide (TVPO); Table S1: Fractional atomic coordinates and equivalent isotropic displacement parameters for TVPO; Table S2: Anisotropic displacement parameters for TVPO; Table S3: Bond angles for TVPO; Table S4: Torsion angles for TVPO; Table S5: Hydrogen atom coordinates and isotropic displacement parameters for TVPO; Figure S2: A 3-D view showing the structure of model compound TVPO.2THF; Figure S3: A 3-D view showing the structure of I1 in the presence of two explicit THF molecules; Figure S4: A potential energy diagram for the reaction between TVPO and VMB; Figure S5: A 3-D view showing the structure of model compound TVPO-Mg(Br)(CHCH_2_); Table S6: Cartesian coordinates for the reaction between TVPO and piperazine (Pip) and vinylmagnesium bromide (VMB).
