# Supplementary material for: Trivinylphosphine Oxide: Synthesis, Characterization, and Polymerization Reactivity Investigated Using Single-Crystal Analysis and Density Functional Theory
Source: Molecules. 2023 Aug 17;28(16):6097. doi: 10.3390/molecules28166097 (PMC10459575; doi:10.3390/molecules28166097)
Supplement: Supplementary file 1 [file molecules-28-06097-s001.zip › molecules-2525054-supplementary.pdf]

Supplementary Materials

Trivinylphosphine Oxide: Synthesis, Characterization, and Polymerization Reactivity Investigated via Single Crystal Analysis and Density Functional Theory

Khalifah A. Salmeia, [1,2,]* Akef T. Afaneh, [1,]* Reem R. Habash,[1] Antonia Neels [3,]*

[1] Department of Chemistry, Faculty of Science, Al-Balqa Applied University, Al-Salt 19117, Jordan

[2] Laboratory for Advanced Fibers, Swiss Federal Laboratories for Materials Science and Technology (Empa), 9014 St. Gallen, Switzerland

[3] Center for X-ray Analytics, Swiss Federal Laboratories for Materials Science and Technology (Empa), Dübendorf 8600, Switzerland

*Corresponding Authors: khalifah.salmeia@bau.edu.jo (K.A.S.); umafaneh@bau.edu.jo (A.T.A.); antonia.neels@empa.ch (A.N.)

Table of Contents

**Figure S1**:NMR Spectra of Trivinylphosphine oxide	S2
**Table S1**: Fractional Atomic Coordinates and Equivalent Isotropic Displacement Parameters for TVPO	S3
**Table S2**: Anisotropic Displacement Parameters for TVPO	S3
**Table S3**: Bond Angles for TVPO	S3
**Table S4**: Torsion Angles for TVPO	S4
**Table S5**: Hydrogen Atom Coordinates and Isotropic Displacement Parameters for TVPO	S4
**Figure S2**: 3-D view of the structure of model compound TVPO.2THF	S5
**Figure S3**: 3-D view of the structure of I1 in the presence of two explicit THF molecules	S5
**Figure S4**: The Potential Energy Diagram for the Reaction of TVPO with VMB	S6
**Figure S5**: 3-D view of the structure of model compound TVPO-Mg(Br)($CHCH_2$)	S6
**Table S6**: Cartesian coordinates for the reaction of TVPO with Piperazine (Pip) or Vinylmagnesium bromide (VMB)	S7-S12

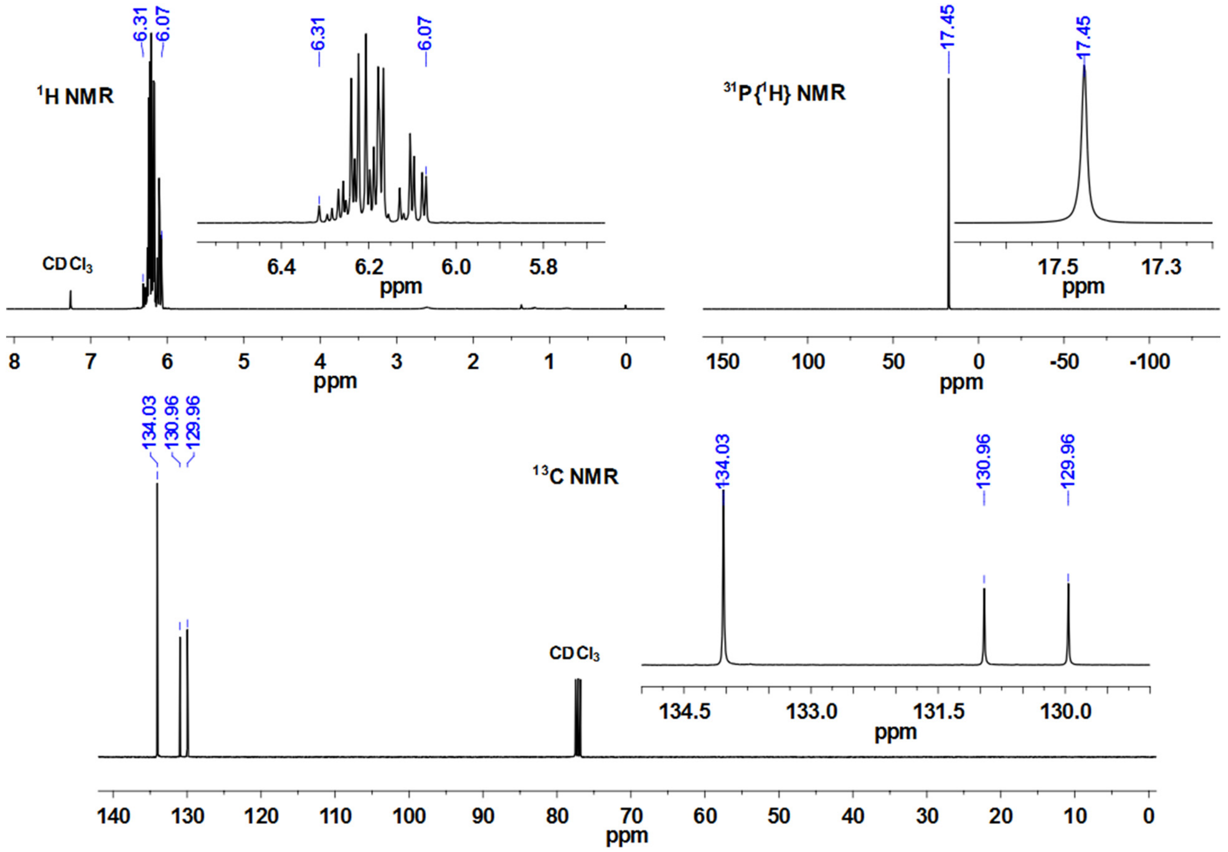

Figure S1: ^{1}H, ^{13}C{^{1}H}, and ^{31}P{^{1}H} NMR spectra (400 MHz, $CDCl_3$) of trivinylphosphine oxide (TVPO)

Table S1: Fractional Atomic Coordinates (×10^4) and Equivalent Isotropic Displacement Parameters (Å^2×10^3) for TVPO. U_{eq} is defined as 1/3 of of the trace of the orthogonalised U_{IJ} tensor.

**Atom**	***x***	***y***	***z***	**U(eq)**
C1	3911(2)	6181.5(16)	3041(2)	32.1(4)
C2	4451(3)	7500	-2025(4)	41.1(7)
C3	4785(3)	7500	-220(4)	33.5(6)
C5	3010(2)	5280.9(18)	3340(3)	41.0(5)
O1	2013.5(17)	7500	900(2)	28.6(4)
P1	3486.4(6)	7500	1611.5(9)	24.5(2)

Table S2: Anisotropic Displacement Parameters (Å^2×10^3) for TVPO. The Anisotropic displacement factor exponent takes the form: $-2\pi^2[h^2a^{*2}U_{11}+2hka^*b^*U_{12}+\ldots]$

**Atom**	**U11**	**U22**	**U33**	**U23**	**U13**	**U12**
C1	32.7(9)	32.7(9)	30.9(9)	2.4(7)	-1.7(8)	5.4(8)
C2	46.2(17)	41.4(15)	35.8(15)	0	8.4(13)	0
C3	30.7(14)	33.6(13)	36.2(14)	0	4.8(12)	0
C5	49.2(12)	32.2(10)	41.7(11)	10.4(8)	-4.7(9)	1.0(9)
O1	23.3(9)	27.1(9)	35.4(9)	0	-4.0(8)	0
P1	24.4(4)	22.7(4)	26.3(4)	0	-0.1(2)	0

Table S3: Bond Angles for TVPO

**Atom**	**Atom**	**Atom**	**Angle/˚**	**Atom**	**Atom**	**Atom**	**Angle/˚**
C5	C1	P1	122.14(15)	C3	P1	C1	104.65(8)
C2	C3	P1	122.4(2)	O1	P1	C1[1]	113.74(7)
C1	P1	C11	105.19(12)	O1	P1	C1	113.74(7)
C3	P1	C11	104.65(8)	O1	P1	C3	113.89(11)

[1]+X,3/2-Y,+Z

Table S4: Torsion Angles for TVPO

A	B	C	D	Angle/°	A	B	C	D	Angle/°
C2	C3	P1	C1	124.81(7)	C5	C1	P1	$C1^1$	128.18(15)
C2	C3	P1	$C1^1$	-124.81(7)	C5	C1	P1	C3	-121.83(18)
C2	C3	P1	O1	0.000(0)	C5	C1	P1	O1	3.1(2)

^{1}+X,3/2-Y,+Z

Table S5: Hydrogen Atom Coordinates ($Å×10^4$) and Isotropic Displacement Parameters ($Å^2×10^3$) for TVPO

Atom	*x*	*y*	*z*	U(eq)
H1	4818.4	6132.44	3602.21	39
H2A	3489.29	7500	-2396.93	49
H2B	5171.16	7500	-2959.75	49
H3	5752.26	7500	124.25	40
H5A	2097.83	5315.47	2788.91	49
H5B	3269.33	4593.46	4106.6	49

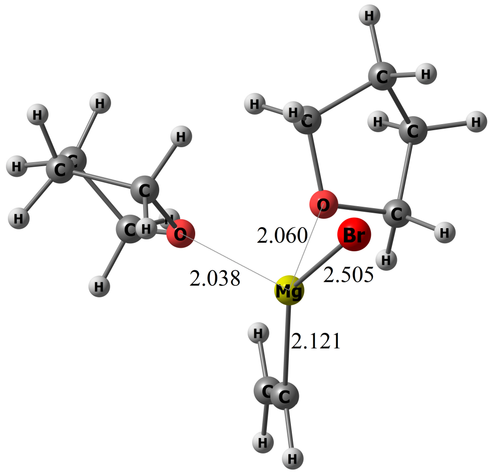

Figure S2: 3-D view of the structure of model compound TVPO.2THF.

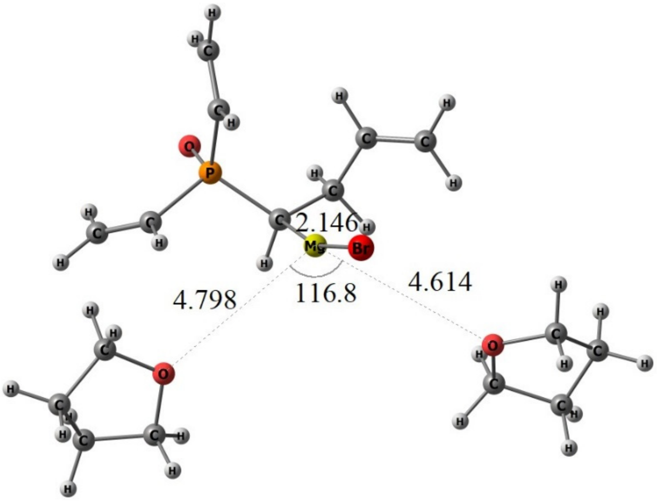

Figure S3: 3-D view of the structure of I1 in the presence of two explicit THF molecules. The distance between the Mg metal ion and the closest THF is about 4.6 Å. The effect of THF on the geometry of I1 is insignificant.

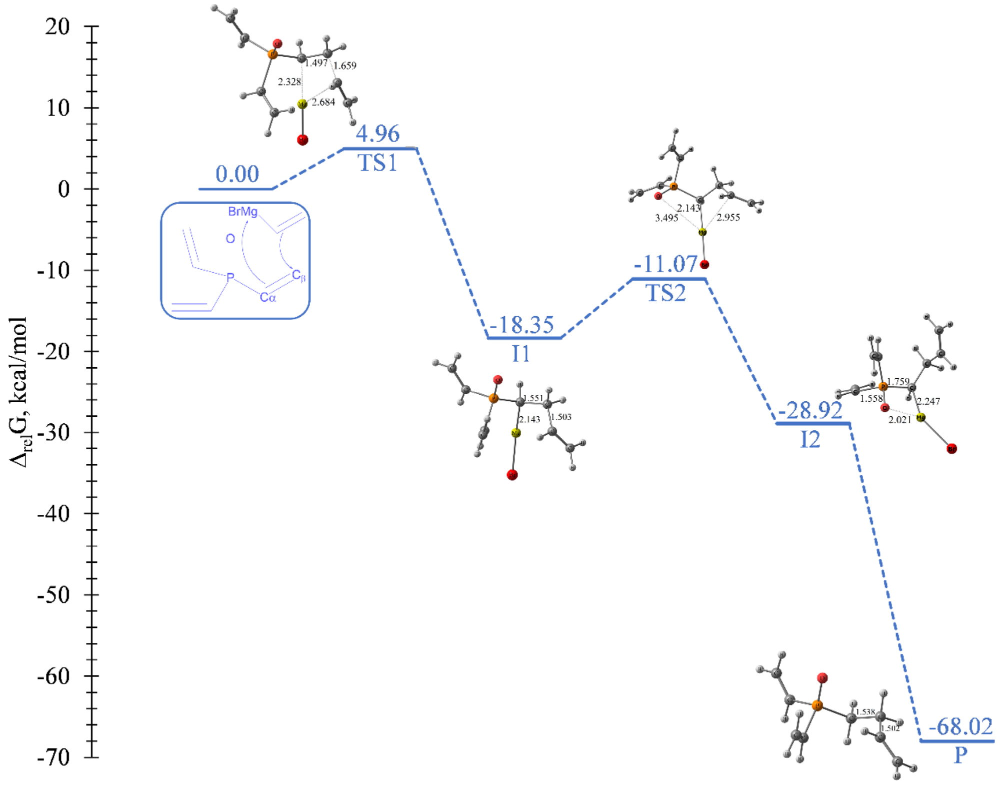

Figure S4: The Potential Energy Diagram for the Reaction of TVPO with VMB. The energies calculated at M062x/6-311++G(d,p) level of theory.

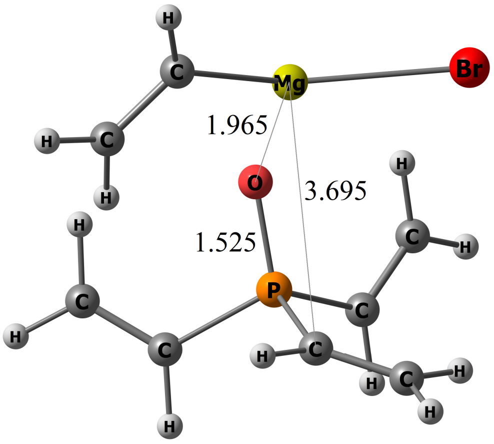

Figure S5: 3-D view of the structure of model compound TVPO-Mg(Br)($CHCH_2$). Less stable by 6.5 kcal/mol than the structure I1 shown in **Figure 3** (blue pathway).

Table S6: Cartesian coordinates for the reaction of TVPO with Piperazine (Pip) or VinyImagnesium bromide (VMB)

**S6a**) Cartesian coordinates for TVPO			
**Atoms (Atomic number)**	**x**	**y**	**z**
6	-1.63975	-0.15682	-0.65514
1	-1.7184	-0.16457	-1.73922
6	1.110538	2.470534	0.125639
1	1.114974	2.485606	1.189904
1	1.525196	3.384219	-0.28577
6	0.67104	1.499762	-0.6539
1	0.704304	1.571083	-1.73803
6	-2.6947	-0.26135	0.121226
1	-2.6763	-0.25983	1.189793
1	-3.69358	-0.36002	-0.28929
8	0.014867	0.014296	1.566887
15	-0.0063	0.005182	0.063814
6	0.951565	-1.32835	-0.65413
1	0.996662	-1.39298	-1.73826
6	1.574787	-2.19237	0.125614
1	1.58442	-2.20382	1.189396
1	2.160753	-3.00711	-0.28539

**S6b**) Cartesian coordinates for TS1 of the reaction of TVPO and Pip (1:1 molar ratio)			
**Atoms (Atomic number)**	**x**	**y**	**z**
6	-3.68942	-0.45027	-0.53582
1	-3.70163	0.032025	-1.51044
6	-1.41193	1.785305	1.614663
1	-1.49994	1.21842	2.536656
1	-1.1505	2.835086	1.69199
6	-1.61125	1.202854	0.437784
1	-1.53213	1.757045	-0.49469
6	-4.79043	-0.96118	0.005093
1	-4.75918	-1.43678	0.980603
1	-5.74684	-0.91934	-0.50446
8	-2.21865	-1.20308	1.686388
15	-2.10894	-0.53273	0.342566
6	-1.01174	-1.38923	-0.83897
1	0.400702	-0.45951	-1.19846
6	0.278074	-1.83741	-0.22536
1	0.12587	-1.9629	0.849501
1	0.54214	-2.82768	-0.6363

6	2.60396	-1.14403	0.409322
6	1.864344	-0.68713	-1.82163
6	3.698877	-0.09153	0.267152
1	3.008325	-2.13412	0.125393
1	2.276153	-1.20331	1.450878
6	2.959206	0.363567	-1.97157
1	2.230179	-1.66253	-2.19528
1	1.00737	-0.39837	-2.43438
1	4.566211	-0.37223	0.868018
1	3.321823	0.864317	0.646765
1	3.284374	0.414389	-3.01264
1	2.545372	1.339945	-1.69822
7	1.454194	-0.79811	-0.42365
7	4.122609	0.112532	-1.1194
1	4.59004	-0.73042	-1.44472
1	-1.56546	-2.2642	-1.19017

**S6c**) Cartesian coordinates for I of the reaction of TVPO and Pip (1:1 molar ratio)			
**Atoms (Atomic number)**	**x**	**y**	**z**
6	-3.45973	0.048589	-0.63188
1	-3.53219	0.226319	-1.7023
6	-0.67299	2.424513	0.550673
1	-0.71625	2.167332	1.60479
1	-0.27522	3.398158	0.285797
6	-1.08738	1.566987	-0.37504
1	-1.05575	1.810544	-1.43456
6	-4.5404	-0.1074	0.125514
1	-4.44825	-0.2815	1.193216
1	-5.53976	-0.06528	-0.29355
8	-1.83077	-0.25356	1.586966
15	-1.80543	-0.02315	0.099169
6	-0.97818	-1.34295	-0.85333
1	-0.86061	-1.00181	-1.88549
6	0.338636	-1.80051	-0.22688
1	0.121175	-2.26278	0.739179
1	0.786156	-2.57936	-0.86902
6	2.387924	-1.14217	0.84239
6	1.769561	-0.12861	-1.23685
6	3.313434	0.031954	1.145151
1	2.963236	-1.94174	0.338287
1	1.989977	-1.5537	1.774109
6	2.695927	1.046157	-0.94199
1	2.311929	-0.89198	-1.82672

1	0.930619	0.222594	-1.8419
1	4.162283	-0.30675	1.74251
1	2.762583	0.773654	1.733677
1	3.092554	1.449208	-1.87601
1	2.114287	1.834778	-0.45305
7	1.269697	-0.70692	0.008373
7	3.811244	0.696479	-0.06079
1	4.429081	0.056343	-0.55414
1	-1.67578	-2.18454	-0.87769

**S6d**) Cartesian coordinates for TS2 of the reaction of TVPO and Pip (2:1 molar ratio)			
**Atoms (Atomic number)**	**x**	**y**	**z**
6	-2.07989	-0.49799	-1.91229
1	-1.27344	-1.11582	-1.52545
6	-1.20755	3.114303	-0.68503
1	-2.06396	3.620637	-1.12025
1	-0.33258	3.711716	-0.45351
6	-1.24294	1.807599	-0.44825
1	-0.39676	1.280595	-0.0133
6	-2.60041	-0.70459	-3.11749
1	-3.40473	-0.07614	-3.48711
1	-2.24276	-1.49847	-3.76394
8	-3.79338	1.624587	-1.54965
15	-2.70894	0.837134	-0.86404
6	-3.19052	-0.00912	0.680465
1	-3.90431	-1.28155	1.663008
6	-2.27991	-1.14588	1.073174
1	-1.94091	-1.65795	0.155546
1	-1.38973	-0.73275	1.556579
6	-2.31752	-3.47509	1.995494
6	-2.82652	-1.63435	3.432484
6	-2.97454	-4.41713	2.988754
1	-1.23324	-3.41979	2.203907
1	-2.45967	-3.8771	0.991105
6	-3.47907	-2.57984	4.425652
1	-1.7693	-1.48872	3.717222
1	-3.32451	-0.66209	3.474609
1	-2.48159	-5.39176	2.944156
1	-4.03365	-4.55651	2.707264
1	-3.34311	-2.17721	5.430981
1	-4.56304	-2.6419	4.218408
7	-2.92586	-2.15031	2.0707
7	-2.86404	-3.90062	4.34862

1	-2.80979	0.690511	1.28833
6	-3.14214	-4.48433	6.756198
1	-3.67852	-3.59364	7.09347
6	0.466734	-5.24868	5.374095
1	0.716615	-4.2204	5.130187
1	1.018605	-6.03513	4.871039
6	-0.48745	-5.52025	6.257696
1	-0.75521	-6.54244	6.515436
6	-3.443	-4.83714	5.299835
1	-4.53665	-4.91611	5.171251
1	-3.02364	-5.82406	5.084092
8	-0.85632	-2.80938	6.769487
15	-1.37203	-4.18566	7.096034
6	-1.19585	-4.57109	8.855758
1	-1.49448	-5.55977	9.194992
6	-0.71755	-3.66003	9.696788
1	-0.42423	-2.67793	9.339008
1	-0.60503	-3.8661	10.75555
1	-3.46481	-5.30987	7.397168

**S6e**) Cartesian coordinates of VMB			
**Atoms (Atomic number)**	**x**	**y**	**z**
6	1.55608	3.428501	0
1	2.486441	2.862015	0
1	1.686234	4.512488	0
6	0.353224	2.834925	0
1	-0.48196	3.544073	0
12	0	0.761701	0
35	-0.43276	-1.64684	0

**S6f**) Cartesian coordinates for TS of the reaction of TVPO and VMB			
**Atoms (Atomic number)**	**x**	**y**	**z**
6	-1.01002	-0.97038	1.104701
1	-0.94824	-2.0634	0.958646
6	-4.41958	-1.59268	-0.82185
1	-5.0628	-0.90098	-0.25687
1	-4.93181	-2.41383	-1.34148
6	-3.09778	-1.42285	-0.85714
1	-2.4478	-2.10473	-1.42418
6	-0.14698	-0.39687	1.969739
1	-0.20239	0.673758	2.217856
1	0.588492	-0.97282	2.549796

8	-3.26132	0.978143	0.806389
15	-2.28601	0.022177	0.024404
6	-0.97742	0.708112	-1.16373
1	-1.28771	0.440348	-2.18637
6	-0.93475	2.199689	-1.04708
1	-1.91709	2.662149	-0.8289
1	-0.55886	2.627809	-1.99329
6	0.129205	2.565475	0.171649
1	-0.30864	2.773061	1.162853
6	1.471659	2.483112	0.047994
1	1.951696	2.35125	-0.93159
1	2.147769	2.673013	0.889844
12	1.023603	0.057404	-0.1677
35	3.038984	-0.91178	-0.18221

**S6g**) Cartesian coordinates for I of the reaction of TVPO and VMB			
**Atoms (Atomic number)**	**x**	**y**	**z**
6	-1.51537	-0.09924	1.64809
1	-0.55959	-0.54863	1.911208
6	-3.25307	-2.60408	-0.71485
1	-4.16811	-2.02816	-0.81697
1	-3.30543	-3.67172	-0.89922
6	-2.11606	-2.00691	-0.37266
1	-1.19287	-2.57059	-0.25915
6	-2.26813	0.496536	2.56735
1	-3.22286	0.935865	2.294502
1	-1.95618	0.564504	3.604138
8	-3.44591	0.408505	-0.22508
15	-2.07649	-0.21581	-0.07841
6	-0.78953	0.394951	-1.15722
1	-1.05898	-0.02184	-2.13691
6	-0.78483	1.942618	-1.26397
1	-1.80719	2.323066	-1.38396
1	-0.23026	2.229363	-2.16218
6	-0.14981	2.607056	-0.07416
1	-0.6775	2.515021	0.873331
6	1.011875	3.25731	-0.10693
1	1.565411	3.371817	-1.03531
1	1.443488	3.69863	0.784086
12	1.194705	-0.30144	-0.74364
35	3.392507	-0.77544	0.2278

**S6h**) Cartesian coordinates for P of the reaction of TVPO and VMB			
**Atoms (Atomic number)**	**x**	**y**	**z**
6	0.298544	1.530273	-0.68674
1	0.142726	1.552809	-1.76239
6	3.387068	-0.74911	-0.04381
1	3.365693	-0.683	1.039678
1	4.325159	-1.02244	-0.51423
6	2.295224	-0.50063	-0.75929
1	2.294082	-0.55954	-1.84485
6	0.193587	2.623704	0.060759
1	0.351627	2.577766	1.134174
1	-0.05654	3.586431	-0.3712
8	0.874965	0.023871	1.564483
15	0.752421	-0.05028	0.067284
6	-0.45191	-1.29112	-0.51928
1	0.062691	-2.25498	-0.47444
6	-1.739	-1.35009	0.320106
1	-1.46381	-1.5693	1.356117
1	-2.34717	-2.18268	-0.04214
6	-2.54458	-0.08271	0.274044
1	-2.11314	0.785034	0.76843
6	-3.72506	0.030472	-0.32636
1	-4.1863	-0.81709	-0.82515
1	-4.26961	0.967513	-0.3355
1	-0.6747	-1.08202	-1.57027
